# Application of machine learning algorithms to predict early childhood development in children aged 24–59 months across three East African countries

**DOI:** 10.1371/journal.pone.0332023

**Published:** 2025-09-12

**Authors:** Tsion Mulat Tebeje, Solomon Hailemariam Tesfaye, Gizaw Sisay, Binyam Tariku Seboka, Getanew Aschalew Tesfa, Daniel Sisay, Mesfin Abebe

**Affiliations:** 1 School of Public Health, College of Health Science and Medicine, Dilla University, Dilla, Ethiopia; 2 Department of Midwifery, College of Health Science and Medicine, Dilla University, Dilla, Ethiopia; Korea University - Seoul Campus: Korea University, KOREA, REPUBLIC OF

## Abstract

**Background:**

Early childhood development (ECD) plays a crucial role in shaping the future development of children and it influences their lifelong outcomes. The Early childhood development index 2030 (ECDI2030) serves as an effective tool for monitoring the overall development of children aged 24–59 months at the population level. This study employed machine learning algorithms to identify the predictors of ECD across three East African countries, using the ECDI2030.

**Methods:**

Data were derived from the Demographic and Health Surveys of Kenya, Mozambique, and Tanzania. Seven supervised machine learning algorithms and an ensemble of the best performing models were utilized to predict ECD. The dataset was randomly divided into 80% training and 20% testing sets. The predictive ability of each machine learning model was evaluated using area under the curve (AUC) and the classification metrics. We used SHapley Additive exPlanations (SHAP) to explain the predictions by interpreting feature importance.

**Results:**

About 57.4% (95% CI = 56.5, 58.3) children were developmentally on track in health, learning, and psychosocial well-being. The ensemble model of extreme gradient boosting and random forest was the best algorithm with accuracy of 66% and AUC of 71%. The top three most important predictors of ECD were child age, media exposure, and maternal education level with a mean absolute SHAP value of +0.17, + 0.12, and +0.1, respectively. The beeswarm plot of SHAP revealed that children aged 24–35 months, those whose mothers were not exposed to media, or those whose mothers had completed at least secondary education were more likely to be developmentally on track.

**Conclusion:**

In East Africa, only the modest majority of children were developmentally on track. Policies should prioritize preprimary education, equitable access, and women’s education to empower mothers and improve parenting practices. Promoting appropriate media use while limiting maternal screen time can enhance children’s developmental outcomes in East Africa and other countries with similar socioeconomic contexts, including most sub-Saharan African countries.

## Introduction

Early childhood development (ECD) sets the foundation for being prepared for school, achieving success in education, contributing to the productivity of a nation, and building social capital [1]. Early childhood is strongly correlated with the rapid development of various abilities, such as physical, motor, cognitive, language, and social skills [2]. Within the first five years of life, children start to actively learn about the environment around them, which is closely tied to the development of their verbal, physical, psychological and perceptual abilities. By the age of five, a child’s brain has reached approximately 90% of its developmental capacity, making these years critical [3,4].

For children to reach their full potential, it is essential to provide them with responsive care, balanced nutrition, nurturing, and a safe environment, which collectively contribute to creating an environment where children can live, learn, grow, and develop optimally [5]. Yet, globally, an estimated 250 million children under the age of five are at risk of not developing to their full potential due to severe poverty and stunting [6]. In low- and middle-income countries (LMICs), approximately one-third of preschool-aged children (80.8 million) do not meet fundamental developmental milestones in cognitive or socioemotional domains, with sub-Saharan Africa (SSA) accounting for the largest share [7]. Poverty, malnutrition, contagious diseases, poor health, psychological issues, and unstimulating environments are the major factors contributing to the lack of progress in children development in LMICs [8–11]. Between 2010 and 2016, 25.3% of children in 63 LMICs experienced a deficit in development, with 42% in West and Central Africa experiencing developmental delay [12]. SSA had the smallest reduction in poor childhood development between 2004 and 2010 and had the highest prevalence of children at risk of poor development. Among the top ten LMICs with the largest number of children at risk were Ethiopia and Tanzania, both located in East Africa [13].

Given the risk of developmental delays, population-level measures can serve as valuable tools for not only quantifying ECD but also predicting social, academic, and emotional well-being later in life [14,15]. This suggests that monitoring and evaluating ECD at a broader scale can offer insights into future outcomes in various domains of well-being. The Early Childhood Development Index 2030 (ECDI2030) is a population-level monitoring tool developed by the UNICEF as a measure of the three domains (health, learning, and psychosocial wellbeing) stated in the sustainable development goal (SDG) indicator 4.2.1 [16]. Target 4.2 aims to ensure that all girls and boys have access to quality early childhood development, care and preprimary education to be ready for primary education by 2030 [17]. Evidence from Palestine and Mexico suggests that the ECDI2030 is a promising and viable tool for monitoring the overall development of children aged 24–59 months at the population level [18]. The demographic and health survey (DHS) collected data and provided insights into the ECDI for children aged 24–59 months to assess their development across the three essential domains [19].

Beyond measurement, countries and organizations have implemented initiatives to enhance ECD, reflecting its importance to national progress. For instance, Kenya rolled out an updated ECD Policy that takes a holistic, integrated approach to supporting children from birth through age eight in 2022 [20]. Similarly, Tanzania launched a national multisectoral ECD program spanning from 2021/22–2025/26 to ensure that all children in the country are developmentally on track by integrating health, nutrition, and simulation interventions to reach their full potential [21]. In addition to country-specific initiatives, the Aga Khan Foundation has been actively contributing to ECD in eleven countries among which three are East African countries: Kenya, Tanzania, and Uganda. The foundation also provides preprimary education to the community, ensuring that children have access to an internationally benchmarked curriculum [22,23]. Moreover, East African Center for Child Neurodevelopment, EACCN, focuses on neurodevelopmental disorders and healthy child development by training adults, creating tools for identifying developmental challenges, advising on effective ECD policies, and supporting the health of pregnant women and young children, mainly in East Africa [24].

Despite the concerted efforts, only 39% and 47% of children aged 24–59 months were developmentally on track in Mozambique and Tanzania, respectively [25,26]. Meanwhile 78% children were developmentally on track in Kenya [27]. This highlights the importance of continued efforts and interventions to ensure that all children reach their full potential. Identifying factors that predict ECD guides effective policies and interventions.

The socioeconomic status of a family, ineffective parenting techniques, a lack of stimulation (such as reading books to kids, giving them educational resources, assisting them with numeracy skills and taking them to museums, zoos or parks [28]), and inadequate nutrition all have a significant impact on ECD [12,29]. To predict ECD and identify the most important determinants, we used a machine learning approach. This study is one of the few [30] that has been conducted to predict ECD through the utilization of machine learning techniques. However, none of the previous studies used the latest measure of childhood development, the ECDI2030. The ECDI2030 comprehensively covers three domains, is age-appropriate, and is a standardized tool that provides population-level data for monitoring SDG 4.2.1 progress, unlike the former ECDI, which was used as a proxy indicator [31]. Therefore, the objective of this study was to determine the predictors of ECD by applying machine learning approaches across three East African countries using the ECDI2030. Our findings could be useful to close evidence gaps, provide up-to-date information on ECD, and develop guidance and programs.

## Methods and materials

### Data Source and population

Data was obtained from the Demographic and Health Surveys’ early childhood development (ECDI2030) module, which were carried out in three East Africa countries (Table 1) [25–27]. The DHS, a population-based cross-sectional study conducted every five years, has been serving as a reliable source of data on various population health concerns in LMICs. DHS sampling designs typically utilize two-stage probability sampling drawn from an existing sampling frame, which is usually based on the most recent census data [32]. Information regarding the DHS methodology can be accessed through the official database: https://dhsprogram.com/methodology/survey-types/DHS-Methodology.cfm. We utilized the Kid’s record dataset (KR file) for this study. The source population included children aged 24–59 months in East Africa. The youngest biological children living with their mothers were included. Ultimately, a total of 12,860 children aged 24–59 months were included in the study.

**Table 1:** Study sample and the prevalence of early childhood development in three East African countries.

Country	Survey year	Frequency		Prevalence of ECD (95% CI)
Unweighted	Weighted
**Kenya**	2022	5,479	4,791	78.1 (76.9, 79.3)
**Mozambique**	2022	2,445	2,635	39.0 (37.2, 40.9)
**Tanzania**	2022−23	4,936	5,022	47.3 (45.9, 48.7)
Total		12,860	12,448	57.4 (56.5, 58.3)

*ECD: early childhood development; CI: confidence interval*

### Variables and measurements

#### Outcome variable.

The dependent variable was early childhood development status calculated by the ECDI2030. The ECDI2030 covers 12 subdomains under three domains of ECD, namely, health, learning, and psychosocial well-being. It contains 20 closed-answer questions or milestones. Mothers were asked how the child behaves in everyday situations and the skills the child has acquired. The first 18 items included a binary yes/no response, and the last two included a graded response. A yes answer to question ECD1-ECD18, any answer other than “daily” to question ECD19, and any answer other than “more” or “a lot more” to question ECD20 was given a score of 1 and a score of 0 otherwise. The mothers who answered “I don’t know” were treated in the same way as those who answered “no”. This gives ECDI2030 a possible range of scores for milestones ranging from 0 to 20. The minimum number of developmental milestones expected for each group is 7, 9, 11, 13, and 15 for ages 24–29, 30–35, 36–41, 42–47, and 48–59 months, respectively. If a child achieves the minimum or greater number of developmental milestones for their age, the child is developmentally on track; if not, they are not on track or are developmentally on delay [33].

#### Predictors.

The independent variables used in this study were child age, child sex, early childhood disease status, vitamin A supplementation status, use of drugs for intestinal parasites, maternal age, maternal and paternal education level, working status, marital status, sex of the household head, internet use, health insurance, residence [34] and sanitation (water and toilet) [35]. To assess a child’s nutritional status, three anthropometric indices, height-for-age z-score (HAZ), weight-for-age z-score (WAZ), and weight-for-height z-score (WHZ), were used. If the HAZ, WHZ, or WAZ was below −2, the child was classified as stunted, wasted, or underweight, respectively, and if their WHZ was greater than +2, they were classified as overweight [32]. Early childhood diseases were classified as “yes” if the child’s mother (or caretaker) stated that the child had symptoms of diarrhea, cough accompanied by short, rapid breathing or difficulty breathing or fever in the past 2 weeks; otherwise, they were classified as “no”. Within the DHS dataset, the composite variable known as the wealth index is classified as “poorest,” “poorer,” “middle,” “richer,” or “richest.” Three categories were used for this study: “poor” (which includes the poorest and poorest people), “medium,” and “rich” (which includes the richest and richest people). Three factors were combined to establish media exposure: listening to the radio, watching TV, and reading newspapers or magazines. A woman is deemed to have had media exposure if she says yes to at least one question. A list of the explanatory variables and their coding can be found in S1 Table.

### Data preprocessing

**Fig 1:**
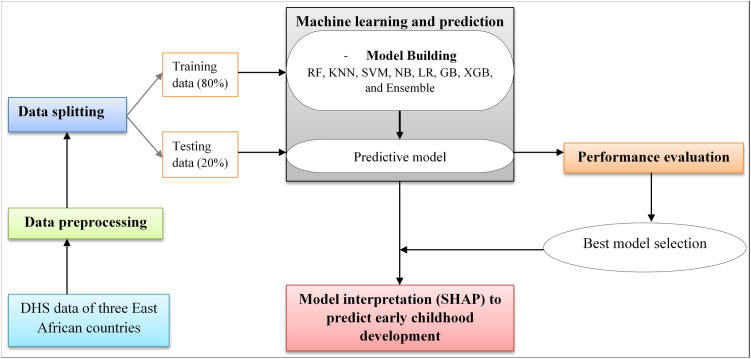
Overview of machine learning framework to predict early childhood development in East Africa.

Each of the three East African countries was assigned a unique code and then combined into a single dataset using the “append using” command in STATA. To ensure the data’s representativeness and account for the sampling design, weights were applied based on the sampling weight. After extracting the data with STATA version 17, the remaining preprocessing and data analysis were carried out using Python 3 (Jupyter Notebook) and its associated libraries. Features with more than 40% missing data were excluded [36] from the machine learning analysis but retained for descriptive analysis: postnatal checkup, birth weight, number of antenatal care visits, stunting, wasting, underweight, overweight, father’s education, and health insurance. All remaining features had complete data and were included in the machine learning analysis. All categorical and string variables were converted to numerical values. To create more balanced datasets for training machine learning models and address class imbalance, various resampling techniques were applied. We employed both oversampling (increasing the number of samples in the minority class) and undersampling (reduces the number of instances in the majority class) approaches [37]. The main stages of our workflow are shown in Fig 1.

### Data splitting and model development

The dataset were randomly divided into training and test sets, constituting 80% (10,288) and 20% (2,572) of the data, respectively. The model was trained on 80% of the sample using five-fold cross-validation to fine-tune the model’s parameters, Then the remaining 20% of the random sample was used to determine how well the model performed [37].

Considering that the problem under study is a classification problem, we employed supervised machine learning approaches [38] to identify children who were developmentally on track from those who were not. In this study, to determine the most effective model for accurately classifying ECD and to identify the key features that significantly contributed to the high level of performance achieved by the selected model, we evaluated the performance of seven different algorithms and an ensemble model: support vector machine (SVM), random forest (RF), Naïve Bayes (NB), logistic regression (LR), K-nearest neighbor (KNN), gradient boosting (GB), extreme gradient boosting (XGB), and an ensemble model. Compared with individual models, ensemble methods combine the predictions of multiple base models or estimators to generate predictions that are more accurate and robust. Therefore, to enhance the classification of ECD, we conducted an ensemble approach by combining the predictions of two top-performing models to assess whether this ensemble strategy would result in improved classification accuracy and yield better performance metrics [39]. To fine-tune the hyperparameters of each ML algorithm, we utilized grid search, which is widely recognized as one of the most popular and commonly employed algorithms for hyperparameter tuning [40,41].

### Model performance metrics

The performance of each model was evaluated and compared with one another. We utilized the sensitivity, specificity, accuracy, AUC-ROC and weighted F1-score to evaluate the performance of the prediction models. Sensitivity refers to the proportion of individuals who have the target condition and receive positive test results by the model (true positives correctly identified by the model) [42].


$$Sensitivity=\frac{TP}{\left(TP+FN\right)}$$ (1)

Specificity represents the proportion of individuals who do not have the target condition and who yield negative test results according to the model (true negatives correctly identified by the model) [42].


$$specificity=\frac{TN}{\left(TN+FP\right)}$$ (2)

The accuracy refers to the proportion of correctly predicted data points out of the total number of data points. It measures the proportion of true results (true positive and true negative) within a given population [43].


$$Accuracy=\frac{\left(TN+TP\right)}{\left(TN+TP+FN+FP\right)}$$ (3)

*Where, TP = true positive, TN = true negative, FP = false positive and FN = false negative.*

The F1-score also assesses the predictive performance of a model and is calculated as the harmonic mean of precision and sensitivity by combining them into single metrics.


$$F1-score=2\times \frac{\left(Precision\times Recall\right)}{\left(Precision+Recall\right)}$$ (4)

Receiver operating characteristic (ROC) curves are graphical tools used to assess the performance of classification models. They depict the trade-offs between the true positive rate and false positive rate across different classification thresholds. The area under the ROC curve (AUC) quantifies the overall performance of a classification model, with higher AUC values indicating better accuracy in distinguishing between positive and negative instances [42].

### Model interpretability

To interpret the predictions of the machine learning approaches, we employed the feature impact approach SHapley Additive exPlanations (SHAP). The SHAP values clarify the contributions of individual features to predict ECD and can be used to analyze the overall impact of features on the model’s output. By sorting the features in descending order based on their average absolute SHAP values, we can identify the influential features that have greater impact on the model’s predictions [44,45].

### Ethical approval and consent to participate

The data were obtained from the Demographic and Health Surveys Program with no personal identifiers. This study was a secondary data analysis of publicly available data from the MEASURE DHS program, and there was no interaction between the researcher and the participants. We obtained permission from the DHS Program to access and use the data for our study. The dataset was downloaded from https://dhsprogram.com/data/available-datasets.cfm.

## Results

### Descriptive characteristics of the participants

Of the total children, more than one-third (4,335; 34.8%) were aged 24–35 months, with a mean age of 40.6 (SD = 10.2) months. The majority (73.7%) had no history of any early childhood disease. About 5,889 (47.3%) and 5,710 (45.9%) of the kids had taken neither vitamin A supplements nor intestinal parasite drugs, respectively, in the previous six months. Regarding nutritional status, about 25.5% were stunted, 4.2% were wasted, 12.0% were underweight, and 1.8% were overweight. Most of the respondents 8,610 (69.2%) were living in rural areas. About 20.2% of the mothers and 18.3% of the fathers did not attain formal education. And 43.9% of the participants were from poor households (Table 2).

**Table 2:** Background characteristics and the prevalence of early childhood development in East Africa.

Variables	Category	Frequency (%)	Prevalence of ECD (children developmentally on track)
Child sex	Male	6,263 (50.3)	55.7
	Female	6,184 (49.7)	59.1
Child age (in months)	24-35	4,335 (34.8)	65.3
	36-47	4,317 (34.7)	56.7
	48-59	3,796 (30.5)	49.2
Birth order	1	3,011 (24.2)	61.8
	2-3	4,976 (40.0)	59.0
	4-6	3,319 (26.6)	54.5
	≥7	1,142 (9.2)	47.6
Preceding birth interval	No preceding birth	3,029 (24.3)	61.8
	<24	1,549 (12.5)	48.5
	≥24	7,870 (63.2)	57.5
Postnatal checkup	No	2,569 (76.4)	62.7
	Yes	795 (23.6)	76.0
Birth weight	High (Macrosomia)	1,010 (23.3)	66.8
	Normal	2,698 (62.2)	68.2
	Low	321 (7.4)	62.0
	Unknown	305 (7.1)	37.6
Vitamin A supplement	No	5,889 (47.3)	51.9
	Yes	6,560 (52.7)	62.3
Drug for intestinal parasite	No	5,710 (45.9)	46.3
	Yes	6,738 (54.1)	66.9
Early childhood disease	No	9,174 (73.7)	54.0
	Yes	3,273 (26.3)	67.1
Cough	No	10,412 (83.6)	54.5
	Yes	2,036 (16.4)	72.3
Diarrhea	No	11,503 (92.4)	56.9
	Yes	944 (7.6)	63.1
Stunting	Normal	6,003 (74.5)	68.5
	Stunted	2,049 (25.5)	49.6
Wasting	Normal	7,797 (95.8)	63.6
	Wasted	342 (4.2)	59.4
Underweight	Normal	7,109 (88.0)	65.4
	Underweight	974 (12.0)	51.2
Overweight	Normal	7,990 (98.2)	63.6
	Overweight	149 (1.8)	57.2
Maternal current age	<20	319 (2.6)	50.8
	20-35	9,284 (74.6)	57.8
	36-49	2,844 (22.8)	56.8
Maternal education level	No education	2,510 (20.2)	36.7
	Primary	6,041 (48.5)	54.3
	Secondary or above	3,897 (31.3)	75.6
Paternal education level	No education	1,886 (18.3)	37.6
	Primary	4,926 (47.8)	53.9
	Secondary or above	3,484 (33.8)	73.3
Marital Status	Single	4,982 (40.0)	54.8
	Married	7,465 (60.0)	59.2
Number of ANC	No ANC visit	344 (10.2)	36.0
	1-3	890 (26.4)	61.7
	≥4	2,134 (63.4)	72.3
Working status	Not working	5,823 (46.8)	51.9
	Working	6,625 (53.2)	62.2
Wealth index	Poor	5,463 (43.9)	46.8
	Medium	2,277 (18.3)	55.5
	Rich	4,708 (37.8)	70.6
Number of household members	2-4	4,049 (32.5)	64.0
	5-7	5,747 (46.2)	56.3
	>7	2,652 (21.3)	49.8
Distance to health facility	Big problem	4,183 (33.6)	49.2
	Not a big problem	8,265 (66.4)	61.5
Covered by health insurance	No	7,378 (96.4)	43.4
	Yes	279 (3.6)	71.4
Water source	Improved	8,353 (67.1)	62.1
	Unimproved	4,095 (32.9)	47.8
Sanitation facility	Improved	7,245 (58.2)	64.6
	Unimproved	5,203 (41.8)	47.3
Sex of HHH	Male	9,302 (74.7)	57.0
	Female	3,146 (25.3)	58.6
Media exposure	Not exposed	5,532 (44.4)	44.2
	Exposed	6,916 (55.6)	68.0
Internet use	No	9,553 (76.7)	49.7
	Yes	2,894 (23.2)	82.9
Residence	Urban	3,838 (30.8)	69.6
	Rural	8,610 (69.2)	52.0

*ANC: Antenatal care; HHH: Household head*

The overall prevalence of early childhood development among East African children was 57.4% (95% CI = 56.5, 58.3) (Table 1). The proportion of ECD increased with an increase in maternal and paternal education levels, household wealth status, birth interval, and antenatal care visits. Having parents with secondary or above education level, being from a wealthy household, no preceding birth interval, and antenatal care visits of four or more times yielded a higher proportion of children developmentally on track than their counterparts. Furthermore, the prevalence of ECD among children who were stunted, wasted, underweight, and overweight were 49.6%, 59.4%, 51.2%, and 57.2%, respectively (Table 2).

### Handling imbalanced data

To prevent biased prediction of the outcome variable, we addressed class imbalance on the training set after data splitting. Our initial approach involved training a random forest model as a baseline and applying various resampling techniques (random over-sampler, synthetic minority oversampling technique, near miss under-sampling, and random under sampling) to assess enhancements in the models’ performance. Despite applying the resampling methods, the highest accuracy was achieved on the baseline model with no resampling (Table 3). This may be attributed to the fact that the outcome variable have low class imbalance (43% vs. 57%) and the applied ML algorithms handled this imbalance better without resampling. Therefore, we proceeded to train the machine learning models on their respective baseline model without resampling.

**Table 3:** Summary of the precision, recall, F1 score and accuracy of each resampling techniques.

Resampling techniques	Performance Metrics			
	Precision (macro average)	Recall (macro average)	F1-score (macro average)	Accuracy
Baseline model (RF)	0.64	0.63	0.63	0.65
Random over-sampler	0.64	0.64	0.63	0.63
Synthetic minority oversampling technique	0.63	0.63	0.62	0.62
Near miss under-sampling	0.64	0.64	0.63	0.63
random under sampling	0.64	0.64	0.63	0.63

*RF: random forest*

### Model building and evaluation

We developed seven supervised ML algorithms and an ensemble model (SVM, RF, NB, LR, KNN, GB, and XGB) to predict ECD. Each algorithm’s performance was evaluated and compared in the test set to select the best predictive model. This approach enhanced understanding the ability of each algorithm to predict children who are developmentally on track in East Africa, allowing for identification of the best model.

**Fig 2:**
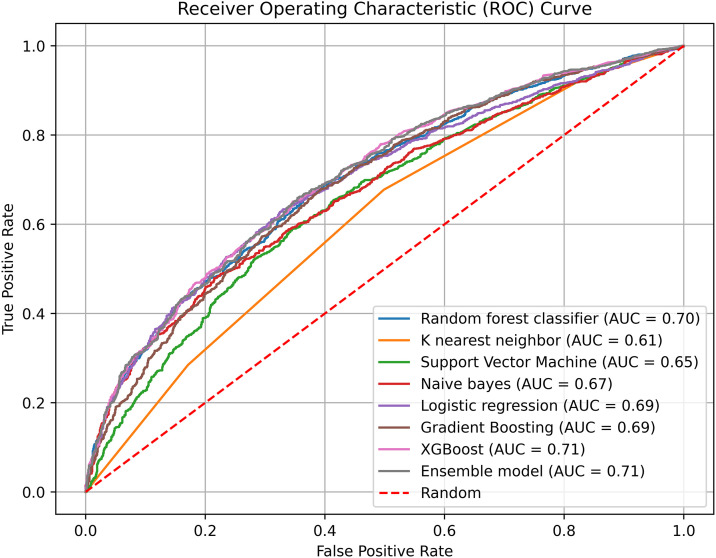
The receiver operating characteristic curve for each machine learning models.

The RF and XGB models were the best predictive models, with an accuracy of 65% and AUC of 70% for RF, and accuracy of 66% and AUC of 71% for XGB. Therefore, we assembled these best-performing models into an ensemble model that resulted in improved performance (accuracy = 66%, sensitivity = 73%, specificity = 0.56, Weighted F1-score = 0.64 and AUC = 0.71) in predicting ECD. This result implies that the ensemble model is 66% correct to predict early childhood development among children aged 24–59 months in East Africa (Table 4). Fig 2 shows the ROC curve and AUC values obtained from each model to classify the ECD. The curves of the XGB and ensemble model show the highest values (AUC = 0.71), which are relatively better at accurately distinguishing ECD status than those of the other algorithms.

**Table 4:** Performance evaluation of the machine learning models to predict early childhood development in East Africa.

Algorithms	Accuracy	Sensitivity (%)	Specificity (%)	Weighted F1-score	AUC-ROC
RF	0.65	76.9	49.0	0.64	0.70
KNN	0.60	67.7	50.1	0.60	0.61
SVM	0.62	79.4	39.4	0.61	0.65
NB	0.61	56.0	58.4	0.61	0.67
LR	0.65	71.9	55.1	0.65	0.69
GB	0.65	73.4	53.7	0.65	0.69
XGB	0.66	78.3	49.5	0.65	0.71
Ensemble (RF + XGB)	0.66	73.1	55.6	0.64	0.71

### Model Interpretability

**Fig 3:**
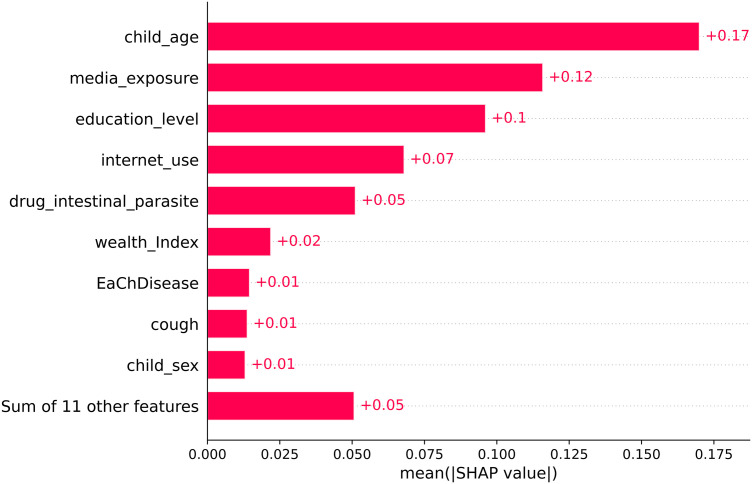
Mean SHAP value of feature importance of early childhood development.

The probability of ECD was estimated using the ensemble model (RF + XGB), and each predictor’s contribution to the expected probability of ECD was evaluated using the SHAP method. The main factors influencing ECD were determined and their contribution was quantified by analyzing the mean absolute SHAP values. The predictors are arranged in descending order of their influence on the prediction of ECD. Child age had the most significant positive impact (+0.17) on predicting ECD. Media exposure (+0.12), maternal education (+0.1), and internet use (+0.07) were the second, third and fourth most influential factors with a positive effect on predicting ECD, respectively. Additionally, taking drugs for intestinal parasites, household wealth index, early childhood diseases, cough, and child sex were among the important predictors of ECD (Fig 3).

**Fig 4:**
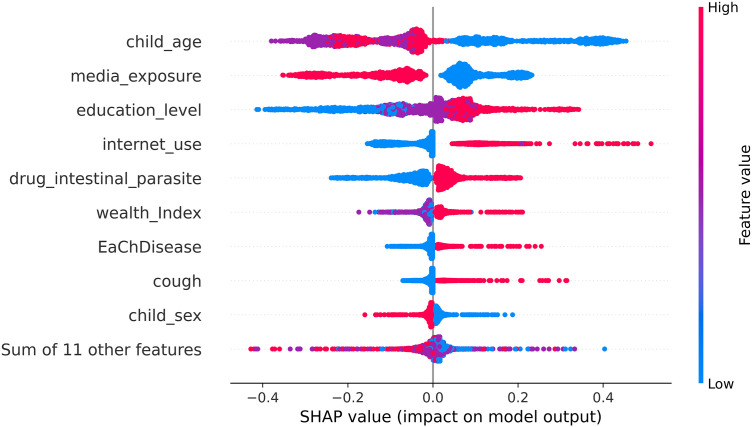
SHAP beeswarm (summary) plot. The y-axis displays the features, and the color represents the value of the feature from low to high (red points represent higher feature values, and blue points represent lower feature values). The x-axis shows the Shapley values for each feature. Each dot signifies the SHAP value of a particular feature for a given data point.

The beeswarm (summary) plot in Fig 4 offers valuable insights into the relationship between features and the ECD. The y-axis indicates the importance of the predictive model, where the features are ranked from most important (top) to least important (bottom). The x-axis shows the influence of a certain feature in the model, with positive SHAP values increase in probability of ECD and negative SHAP values decrease in probability of ECD. Feature values are represented with dots of distinct colors. Red dots denote high category value for that feature and blue dots denote low category value for that feature. For example, a low value of child age (24–35 months) and media exposure (not exposed); and a high value of education level (secondary or above) and internet use (being internet user) are associated with an increased likelihood of being developmentally on track, as shown by the positive SHAP values in the x-axis of the Beeswarm plot (Fig 4). Details on the encoding and what each categorical variable represents can be found in S1 Table.

Child age, maternal education, internet use, and media exposure demonstrated the largest absolute minimum and maximum SHAP values. Maternal education level had a minimum SHAP value of −0.40, meaning that lower education levels were associated with a 40% decrease in the probability of ECD, while higher education levels increased ECD by 35%. Similarly, maternal media exposure showed a minimum SHAP value of −0.35, suggesting that media exposure was linked to a 35% decrease in the probability of ECD, whereas not exposed to media increased the probability by 23%. Child age had a minimum and maximum SHAP values of −0.37 and 0.44, respectively. This implies that older child age is associated with a 37% decrease in the likelihood of being developmentally on track, whereas younger age is associated with a 44% increase (Table 5).

**Table 5:** The minimum and maximum SHAP values for the ensemble model’s features.

Feature	Minimum SHAP	Maximum SHAP
Residence	−0.094	0.216
Sex of HHH	−0.040	0.032
Internet use	−0.152	0.515
Child sex	−0.137	0.189
Child age (in months)	−0.370	0.438
Birth order	−0.198	0.122
Preceding birth interval	−0.174	0.086
Vitamin A supplement	−0.189	0.157
Drug for intestinal parasite	−0.245	0.210
Diarrhea	−0.032	0.021
Cough	−0.069	0.259
Early childhood disease	−0.116	0.229
Maternal current age	−0.031	0.102
Maternal education level	−0.404	0.347
Marital Status	−0.142	0.075
Working status	−0.154	0.124
Wealth index	−0.177	0.233
Distance to health facility	−0.076	0.170
Number of household members	−0.222	0.224
Media exposure	−0.353	0.235

*SHAP: SHapley Additive exPlanations; HHH: Household head*

## Discussion

This study employed machine learning algorithms to predict early childhood development in East Africa in children aged 24–59 months. We trained, tested, and evaluated seven supervised machine learning algorithms: SVM, RF, NB, LR, KNN, GB, and XGB, and an ensemble model (XGB and RF). The accuracy of algorithms for classifying ECD ranges from 60% to 66%. The machine learning models were evaluated to predict whether a child will be developmentally on track or not on track. The results showed that the ensemble model performed better than the other models. The AUC-ROC of the ensemble model was found to be 0.71. This is slightly higher than the AUC of 0.67 reported by Hossain et al. for ML-based predictive modeling of ECD among children aged 36–59 months in Bangladesh [30]. We evaluated the importance of variables that lead to improved performance in the prediction of ECD using the ensemble model. The child’s age, maternal education level, media exposure, and wealth index were found to be important predictors of ECD in this study. A study from Rwanda that used a machine learning technique called classification and regression trees to predict ECD revealed that exposure to any violent discipline, nutritional adequacy, wealth index, sex of a child, adequacy of care, and having toys play a critical role in the development status of a child [46].

To interpret our best performing model, which is the ensemble model, we utilized SHAP. When machine learning and SHAP are used together, they can be used to uncover key features and investigate useful links from data. By calculating each feature’s contribution, it seeks to explain the prediction of an instance [47,48]. Hence, the SHAP approach was used to identify the most important factors influencing ECD. The three most important factors identified and ranked high were child age, media exposure, and maternal education level.

Children aged 24−35 months were more likely to be developmentally on track. While those aged 36−47 and 48−59 months had a higher risk of being developmentally not on track. This finding is unsupported by studies from Bangladesh [30,34], which revealed that children aged 48−59 months were more developmentally on track. This can be explained by the fact that ECD is a continuous process throughout the first five years of life, with various developmental milestones appearing at various ages. While the brain undergoes the majority of its neuron development from birth to the age of three, early childhood education serves as a fundamental cornerstone for a child’s future achievements. The period between 36 and 59 months is a crucial phase in a child’s life. During this time, they start attending daycare or school, which serves as a platform for them to acquire a variety of skills that ensure that children are well prepared for the next phase of their lives [49]. However, in Eastern and southern Africa, the gaps in pre-primary education attendance favors children from richest wealth quintile and urban residence [50] and the net enrollment rate in preprimary education is relatively low. For instance, Tanzania’s net enrollment rate in preprimary education increased only by 9.5 percentage points between 2014−15 (25.9%) and 2020−21 (35.4%), which is far less than the 61% global gross preprimary enrollment rate in 2020 [51,52]. This made children in East Africa unable to fulfill the minimum number of developmental milestones expected for their age.

Children of mothers with an education level of secondary education or above were more likely to be developmentally on track, whereas when mothers are uneducated or have completed only primary education, their children are at greater risk of being developmentally on delay. This is aligned with studies from Bangladesh [30,34], Nigeria [53], and Turkey [54]. A recent study from Uganda found out that maternal education improves parenting through engaging in stimulating activities with children, attending early childhood education programs, and minimizing harsh punishment [55]. Mothers with higher education also have fewer children with birth spacing, more educated partners and higher incomes, give birth in formal medical facilities, prioritize prenatal care from authorized sources, use contraception, and engage in work [56].

This study also showed that children of mothers who are not exposed to media are more likely to be developmentally on track. On the other hand, maternal media exposure increases the risk of being developmentally on delay. This can be explained by mothers who exhibited high levels of media use were more likely to have children with high screen times [57]. This leads to lower communication, interaction, and play and that might result in hyperactivity and lack of attention which reduces children’s ability to learn [58,59]. In Africa, millions of people now have access to television due to the massive expansion of media outlets. However, studies from Bangladesh [30,34,60] found that children from households with media exposure are more likely to be on track of ECD, contradicting our finding. This inconsistency could be resulted from contextual differences in the nature and use of media and the different measures to measure ECD: their study relied on the former ECDI while our study used the new ECDI2030.

### Strengths and limitations

A countrywide representative sample was used for the study, allowing the results to be generalized to the whole population. Another strength is the application of interpretable machine learning models. The study’s limitations should be taken into account when interpreting the results. Since we used secondary data, the study was limited to features presented in the DHS. This lack of information has led to certain aspects of early childhood development being overlooked. The study’s relatively lower prediction power and AUC-ROC might be improved by incorporating those features. We were unable to show a real cause-and-effect relationship because of the cross-sectional nature of the data.

## Conclusion

Early childhood development is the foundation for the growth of a child and a crucial base of a family and a community. However, only a modest majority of children aged 24–59 months in East Africa were developmentally on track. To identify predictors of ECD, machine learning algorithms were implemented. Ensemble of the random forest and extreme gradient boosting achieved better predictive power. The SHAP method of the ensemble model showed that child age, maternal media exposure, and maternal education level were the three highly influential features in predicting ECD. Therefore, prioritizing childhood development specifically among pre-school aged children through preprimary education by increasing the enrollment rate and implementing policies that promote equity is important. Women’s education should also be given priority. Education empowers mothers within households and positively influences parenting practices. Although promoting educational and child-friendly content is beneficial, mothers should limit their own screen time. This helps reduce excessive screen time and media exposure for their children, ultimately fostering better developmental outcomes. These recommendations for ECD are relevant across similar socioeconomic contexts, including other sub-Saharan Africa countries. Overall, this study offers insights with important implications for child health development and policy. It also establishes the basis for evidence-based strategies to increase children who are developmentally on track.

## Supporting information

S1 Table Features and their coding. (DOCX)

S1 Syntax (ZIP)

## Abbreviations

AUC: Area under the curve
AUC-ROC: Area under the curve of receiver operator characteristics curve
EAs: Enumeration areas
ECD: Early Childhood Development
ECDI: Early Childhood Development Index
SDG: Sustainable Development Goal
GB: Gradient boosting
KNN: K-nearest neighbor
LMICs: Low- and middle-income countries
LR: Logistic regression
ML: Machine Learning
NB: Naïve Bias
RF: Random forest
SHAP: SHapley Additive exPlanations
SSA: Sub-Saharan Africa
SVM: Support Vector Machine
XGB: Extreme gradient boosting.
