# Supplementary material for: Application of machine learning algorithms to predict early childhood development in children aged 24–59 months across three East African countries
Source: PLoS One. 2025 Sep 12;20(9):e0332023. doi: 10.1371/journal.pone.0332023 (PMC12431311; doi:10.1371/journal.pone.0332023)
Supplement: S1 Table — (DOCX) [file pone.0332023.s001.docx]

**Application of machine learning algorithms to predict Early Childhood Development among children aged 24-59 months in East Africa**

# **Supplementary Table**

**S1 table: Features and their coding**

| Explanatory variables | Measures | Explanatory variables | Measures |
| --- | --- | --- | --- |
| child age | 24-35=0, 36-47=1, 48-59=2 | marital status | single=0, married=1 |
| child sex | male=1, female=0 | sex of household head | male=1, female=0 |
| early childhood disease | yes=0, no=1 | Residence | urban=1, rural=0 |
| vitamin a supplement | no=0, yes=1 | number of ANC | no=0, 1-3=1, ≥4=2 |
| taking drugs for intestinal parasites | no=0, yes=1 | distance to the health facility | big problem=1,  not a big problem=0 |
| stunted | normal=0, stunted=1 | wealth index | poor=0, medium=1, rich=2 |
| wasted | normal=0, wasted=1 | media exposure | Not exposed=0, exposed=1 |
| underweight | normal=0, underweight=1 | water facility | improved=0, unimproved=1 |
| overweight | normal=0, overweight=1 | toilet facility | improved=0, unimproved=1 |
| birth order | 1=0, 2-3=1, 4-6=2, ≥7=3 | maternal age | <20=0, 20-35=1, 36-49=2 |
| preceding birth interval | <24=, ≥24= | maternal educational level | no=0, primary=1, secondary or above=2 |
| cough | no=0, yes=1 | diarrhoea | no=0, yes=1 |
| postnatal checkup | no=0, yes=1 | paternal educational level | no=0, primary=1, secondary or above=2 |
| Internet use | no=0, yes =1 | working status | no=1, yes=0 |

*ANC: antenatal care*
